# Linking CD11b^**+**^ Dendritic Cells and Natural Killer T Cells to Plaque Inflammation in Atherosclerosis

**DOI:** 10.1155/2016/6467375

**Published:** 2016-03-09

**Authors:** Miche Rombouts, Rachid Ammi, Ilse Van Brussel, Lynn Roth, Benedicte Y. De Winter, Sven R. Vercauteren, Jeroen M. H. Hendriks, Patrick Lauwers, Paul E. Van Schil, Guido R. Y. De Meyer, Erik Fransen, Nathalie Cools, Dorien M. Schrijvers

**Affiliations:** ^1^Laboratory of Physiopharmacology, Faculty of Pharmaceutical, Biomedical and Veterinary Sciences, University of Antwerp, 2610 Antwerp, Belgium; ^2^Laboratory of Experimental Medicine and Pediatrics, Faculty of Medicine and Health Sciences, University of Antwerp, 2610 Antwerp, Belgium; ^3^Hospital Network Antwerp, 2020 Antwerp, Belgium; ^4^Department of Thoracic and Vascular Surgery, University Hospital Antwerp, 2650 Antwerp, Belgium; ^5^StatUa Center for Statistics, University of Antwerp, 2650 Antwerp, Belgium; ^6^Laboratory of Experimental Hematology, Vaccine and Infectious Disease Institute, Faculty of Medicine and Health Sciences, University of Antwerp, 2650 Antwerp, Belgium

## Abstract

Atherosclerosis remains the leading cause of death and disability in our Western society. To investigate whether the dynamics of leukocyte (sub)populations could be predictive for plaque inflammation during atherosclerosis, we analyzed innate and adaptive immune cell distributions in blood, plaques, and lymphoid tissue reservoirs in apolipoprotein E-deficient (ApoE^−/−^) mice and in blood and plaques from patients undergoing endarterectomy. Firstly, there was predominance of the CD11b^+^ conventional dendritic cell (cDC) subset in the plaque. Secondly, a strong inverse correlation was observed between CD11b^+^ cDC or natural killer T (NKT) cells in blood and markers of inflammation in the plaque (including CD3, T-bet, CCR5, and CCR7). This indicates that circulating CD11b^+^ cDC and NKT cells show great potential to reflect the inflammatory status in the atherosclerotic plaque. Our results suggest that distinct changes in inflammatory cell dynamics may carry biomarker potential reflecting atherosclerotic lesion progression. This not only is crucial for a better understanding of the immunopathogenesis but also bares therapeutic potential, since immune cell-based therapies are emerging as a promising novel strategy in the battle against atherosclerosis and its associated comorbidities. The cDC-NKT cell interaction in atherosclerosis serves as a good candidate for future investigations.

## 1. Introduction

Increasing evidence from animal and human studies points to a nonnegligible role for the innate and adaptive immune system in the development of atherosclerosis, still the leading cause of cardiovascular morbidity and mortality in our Western society [1–4]. In fact, it is estimated that approximately 30% of all deaths worldwide can be attributed to cardiovascular disease [5]. Therefore, there is a great need for the discovery of new biomarkers that may help in the early detection of patients at risk as well as the development of new therapies leading to the stabilization or regression of atherosclerotic plaques.

Recent findings suggest that a mismatch in the distribution, phenotype, and/or function of dendritic cells (DC), the main orchestrators of the immune response, contributes to the susceptibility to and the course of atherosclerosis [6–10]. There are two major subpopulations of DC, namely, conventional DC (cDC) and plasmacytoid DC (pDC). In mice, cDC subtypes comprise cDC type 1 (cDC1), encompassing lymphoid-tissue-resident CD8*α* ^+^ cDC and their migratory counterparts CD103^+^ cDC, and CD11b^+^ cDC2 [8, 10, 11]. The specific and highly conserved transcription factor* Zbtb46* can be used to distinguish cDC and their progenitors from other immune cells [12]. Mouse atherosclerotic plaques contain both subtypes of cDC, but CD11b^+^ cDC are most abundant and have been shown to rapidly increase during atherogenesis. Moreover, they are described to promote atherosclerosis [13, 14]. In contrast, CD103^+^ cDC protect against atherosclerosis as they have been shown to support the homeostasis of regulatory T cells (Tregs) in a mouse model of atherosclerosis [15]. In humans, cDC are also segregated into two subtypes, namely, BDCA-3^+^ cDC (cDC1) and BDCA-1^+^ cDC (cDC2) [8, 10]. In addition to cDC, few pDC can be detected in murine and human atherosclerotic lesions, though their exact role in the disease process is still a subject of discussion [16–19].

Murine and human unstable plaques are associated with increased levels of T cells. Activated CD4^+^ effector and memory T cells with a T helper (Th) 1 profile are among the earliest cells to accumulate in atherosclerotic lesions [20]. Natural killer T (NKT) cells represent another subset of T cells that recognize glycolipid antigens presented on CD1d molecules (on antigen-presenting cells) and share surface receptors in common with NK cells. In the past few years NKT cells have become of great interest given the fact that lipid accumulation is a prominent aspect of atherogenesis. Moreover, bidirectional interactions between NKT cells and DC ensure amplification and control of the subsequent innate and adaptive immune responses. Most of the experimental data from animal models attribute a proatherogenic role to NKT cells [21–23]. In humans, however, the pathophysiological role of these cells is less clear.

Although multiple immune cells are involved in atherosclerosis, most studies focus on a single cell type due to technical limitations. Detailed immune cell phenotyping requires the use of multilaser flow cytometers [24]. We previously described a protocol and a gating technique to identify and isolate immune cells from human atherosclerotic plaques using multiparametric flow cytometry [14]. In this study, local and systemic immune cell distributions in murine and human atherosclerosis were characterized simultaneously using flow cytometry and real-time qPCR. The distribution of DC (subsets), NK(T) cells, T cells, and monocytes/macrophages was analyzed both in blood and plaques. Previous research has shown that disturbed flow, caused by carotid ligation, induces rapid and dynamic leukocyte accumulation in the arterial wall [25]. However, adaptive immunity may not be solely driven from within the plaque but may also be driven from plaque-draining lymph nodes or even the periphery (e.g., the spleen). Therefore, possible associations between all the compartments were investigated. Additionally, we assessed the expression of different chemokine receptors during disease development to determine whether the homing functionality of immune cells correlates with changes in immune cell dynamics or plaque development.

## 2. Materials and Methods

### 2.1. Mice

Male and female ApoE^−/−^ mice were fed a Western-type diet (WD, 4021.90, AB Diets) starting at an age of 6 weeks (wk). Mice were sacrificed with sodium pentobarbital (250 mg/kg, i.p.) before onset of atherosclerosis (0 wk of WD) or after 6, 12, and 24 wk of WD. These time points represent healthy artery, fatty streak, fibroatheroma, and advanced atherosclerotic plaques in mice. Analysis of total plasma cholesterol was performed by using a commercially available kit (Randox) following the manufacturer's instructions. Age-matched nonatherosclerotic C57BL/6J control mice on chow feeding were used to adjust for changes related to ageing rather than atherosclerosis. The animals were housed in a temperature-controlled room with a 12-hour light/dark cycle and had free access to water and food.

### 2.2. Patients

To characterize the immune cells in human atherosclerosis, 72 patients that were eligible for endarterectomy at the carotid (*n* = 35; 49%) and femoropopliteal level (*n* = 37; 51%) were recruited from the clinical departments of Thoracic and Vascular Surgery of the Antwerp University Hospital and ZNA Middelheim. From 57 (79%) of the included patients, peripheral blood samples were collected as well to study the systemic immune cell distribution. High sensitivity C-reactive protein (hs-CRP) levels were measured in serum from 35 patients by the clinical lab of the Antwerp University Hospital. Patient characteristics are shown in Table 1.

### 2.3. Ethics Statement

The mouse protocols were approved by the Antwerp University Ethics Committee on Animal Experiments (permit number: 2013-68). The animals received human care and were treated according to the national guidelines for animal protection, and the “Guide for the Care and Use of Laboratory Animals” (National Institutes of Health, 1985). Protocols involving patients were approved by the local Ethics Committee (number 12/25/212), and all research was based on written informed consent with proper arrangements for the protection of the confidentiality of personal data of the individuals concerned.

### 2.4. Cell Isolation and Flow Cytometry from Murine Blood and Tissues

**Figure 1:**
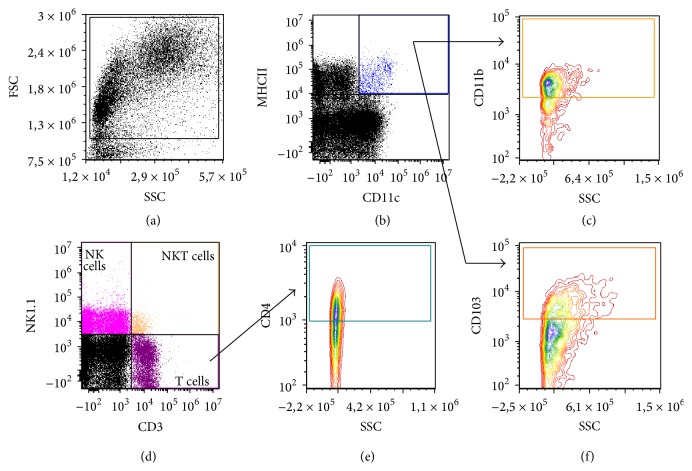
Gating strategy for the analysis of immune cells in murine atherosclerosis. Gates are set on isotypes to correct for nonspecific binding. (a) Plots are pregated on FSC and SSC to define the total percentage of leukocytes from cell debris. (b) The total cDC population was identified based on the expression of CD11c and MHCII. (c, f) Based on their expression of CD11b (c) and CD103 (f) two cDC subsets were identified. A distinction was made between circulating Ly-6C^high^ and Ly-6C^low^ monocytes and tissue resident macrophages (plots not shown). (d) Lymphocyte subsets were identified as T cells (CD3^+^NK1.1^−^), NK cells (CD3^−^NK1.1^+^), and NKT cells (CD3^+^ NK1.1^+^). (e) Th cells were defined as CD4^+^ cells within the total T cell population.

After sacrificing the mice, blood was obtained by cardiac puncture. Single cell suspensions of the aorta draining mediastinal lymph nodes (LN) [26] and the spleen were prepared by passage through a 40 *μ*m cell strainer. Erythrocytes were lysed using a red blood cell lysing buffer (Hybri-Max, Sigma-Aldrich). Remaining leukocytes were counted using a hemocytometer and labelled with anti-mouse monoclonal antibodies (Supplemental Table S1 in Supplementary Material available online at http://dx.doi.org/10.1155/2016/6467375) at 4°C in FACS buffer (PBS + 0.1% BSA (Sigma-Aldrich) + 0.05% NaN_3_ (Merck)) in the presence of CD16/32 Fc-receptor blocker (BioLegend). Cells were analyzed on a BD Accuri C6 cytometer (BD Biosciences). Debris and dead cells were excluded based on forward scatter, side scatter, and positive staining for propidium iodide (Life Technologies). The gating strategy is depicted in Figure 1. Data analysis was performed with FCS Express 4 (De Novo Software).

### 2.5. Cell Isolation and Flow Cytometry from Human Atherosclerotic Plaques and Peripheral Blood

Atherosclerotic plaques were collected in RPMI 1640 medium (Life Technologies) and kept at room temperature until processing. Cell isolation was performed as described [14]. Briefly, within 2 h after surgery the plaque specimens were dissected into small pieces, followed by an enzymatic digestion with 2.5 mg/mL collagenase IV (Life Technologies) and 0.2 mg/mL DNase I (Roche) for 2 h at 37°C. After digestion, the residue was filtered over a 40 *μ*m cell strainer. PBMC were isolated from blood samples by Ficoll (GE Healthcare) density gradient centrifugation. After isolation, cells from plaque and blood were blocked with mouse gamma globulins and stained with an optimized 9-color panel of mouse anti-human monoclonal antibodies (Table S1). To eliminate the abundance of cell debris and extracellular lipids in the digested plaque suspensions, we used a gating strategy as described previously [14]. All measurements were performed on the FACSAria II (BD Biosciences). Data acquisition and analysis were done using FACSDiva 6.1.2 (BD Biosciences).

### 2.6. Gene Expression Analysis

The aortic adventitia of mice was partially digested and removed from the rest of the vessel following incubation in an enzyme digestion solution composed of 781.25 U collagenase II and 14.0625 U elastase (Worthington) in 2.5 mL PBS for 10 minutes at 37°C [27]. Total RNA was extracted from the aorta, stripped from the adventitial layer, using a TRIzol-based RNA isolation protocol (Ambion). RNA concentrations were measured with a NanoDrop ND-1000 spectrophotometer (NanoDrop Technologies). RNA was reverse-transcribed with the SuperScript II Reverse Transcriptase kit (Life Technologies). Quantitative gene expression analysis was performed on a 7300 Real-Time PCR System (Applied Biosystems) using SYBR green technology (SensiMix, GC Biotech). The parameters for PCR amplification were 50°C for 2 min and 95°C for 10 min, followed by 40 cycles of 95°C for 15 s, 60°C for 1 min, and 72°C for 30 s. Melting curves were checked for amplification of a single, specific product. Used primer pairs are summarized in Table S2. All data were analyzed using qBase+ 3.0 (Biogazelle).

### 2.7. Histological Analysis

After sacrificing ApoE^−/−^ mice, the proximal ascending aorta and brachiocephalic artery were collected, embedded in Neg-50 (Thermo Scientific), and snap-frozen in liquid nitrogen. Atherosclerotic plaque size, stenosis, and necrotic core (acellular area with a threshold of 3000 *μ*m^2^) were analyzed on haematoxylin-eosin (H-E) stained 5 *μ*m cryosections (Table 2). All images were acquired with Universal Grab 6.1 software using an Olympus BX40 microscope and were quantified with ImageJ software (National Institutes of Health).

### 2.8. Statistical Analysis

All data are presented as mean ± SEM. Multiple comparisons of means were performed for the analysis of all mouse data using one-way ANOVA followed by Dunnett's Multiple Comparison test or two-way ANOVA followed by Bonferroni's Multiple Comparison test where appropriate. Differences between human plaques derived from the carotid and femoral artery were tested with the independent Student's *t*-test. Variables that failed normality were logarithmically transformed or analyzed with the nonparametric Mann-Whitney *U* test. Correlations between local and circulating cells in atherosclerosis were described using Spearman's rank-order correlation coefficient. Statistical analysis was performed using Prism 5.0 (GraphPad) or R version 3.1.2 [28]. *P* < 0.05 was considered statistically significant.

## 3. Results

### 3.1. Analysis of Immune Cells in Blood, Plaque, and Lymphoid Tissue of ApoE^−/−^ Mice

**Figure 2:**
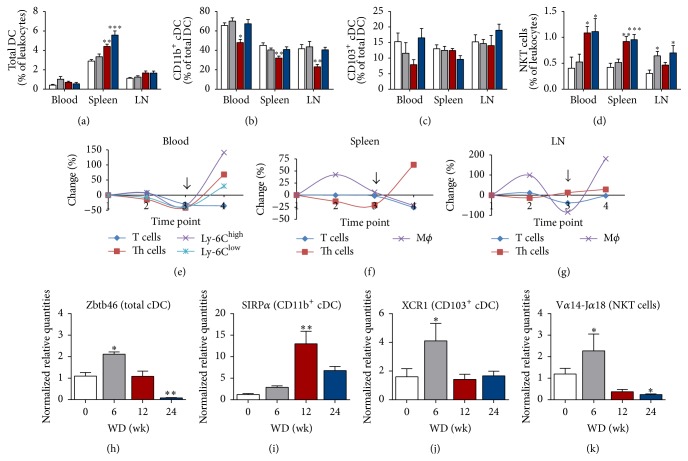
Flow cytometry and gene expression results of leukocyte(s) (subsets) in ApoE^−/−^ mice during atherogenesis. Bar graphs representing mice sacrificed after 0 wk (white bars, *n* = 7–12), 6 wk (grey bars, *n* = 9–12), 12 wk (red bars, *n* = 9-10), and 24 wk (blue bars, *n* = 10–12) of WD. (a) Percentages of the total DC population. (b) Percentages of the CD11b^+^ cDC subset within the total DC population. (c) Percentages of CD103^+^ cDC within the total DC population. (d) Percentages of NKT cells within the total leukocyte population. (e–g) Graphs showing fluctuations (as percentage change over time) in T cells, Th cells, and monocytes/macrophages in blood (e), spleen (f), and mediastinal LN (g) at different time points (1 = 0 wk, 2 = 6 wk, 3 = 12 wk, and 4 = 24 wk of WD). The arrow at time point 3 ( = 12 wk of WD) indicates dramatic leukocyte changes in all compartments. (h–k) Normalized expression levels of Zbtb46 (h), SIRP*α* (i), XCR1 (j), and V*α*14-J*α*18 (k) mRNA in aortic tissue samples; ^*∗*^ *P* < 0.05, ^*∗∗*^ *P* < 0.01, and ^*∗∗∗*^ *P* < 0.001.

During atherosclerotic plaque development in ApoE^−/−^ mice the percentage of total DC increased in the spleen but no distinct changes were observed in blood or LN (Figure 2(a)). Interestingly, at all locations and time points, CD11b^+^ cDC (or cDC2) represented the most predominant subset. Furthermore, a significant drop of this cDC subset was seen at all locations after 12 wk of WD (Figure 2(b), red bars). Regarding the other cDC subset, CD103^+^ cDC or cDC1, we found no significant difference over time (Figure 2(c)). No significant changes were seen in the frequency of NK cells (data not shown). Additionally, the percentage of Ly-6C^low^ resident monocytes and their inflammatory counterparts, Ly-6C^high^ monocytes, in blood is decreased after 12 wk of WD. The percentage of both subtypes increased between 12 and 24 wk of WD, which was most pronounced in the Ly-6C^high^ subset (Figure 2(e)).

Regarding cells of the adaptive immunity, the percentage of T cells gradually declined in all studied compartments. The reduction in T cells was most pronounced after 24 wk of WD in blood, spleen, and mediastinal LN compared with mice sacrificed before the onset of atherosclerosis (Figures 2(e)–2(g)). With regard to T cell subsets, the percentage of total CD4^+^ Th cells significantly decreased after 12 wk of WD in blood and spleen as compared to mice at 0 wk of WD (Figures 2(e) and 2(f)). Interestingly, percentages of NKT cells, known as T lymphocytes with innate effector functions, are increased after 12 wk (in blood and spleen) and 24 wk of WD (in blood, spleen, and LN) (Figure 2(d)). All graphs indicate a clear turning point in immune cell dynamics at 12 wk of WD (Figures 2(e)–2(g), arrows). At this time point, a substantial increase was observed in the size and areas of the atherosclerotic plaques in the brachiocephalic and proximal aorta (Table 2). Between 12 and 24 wk of WD systemic immune activation is induced as evidenced by an increase in the majority of immune cells above baseline in all locations investigated.

Expression levels of DC (subset) genes within plaque-containing aortic tissues were measured with qPCR. The expression of Zbtb46, a transcription factor used to distinguish the total cDC population from other immune cells, was increased after 6 wk of WD, returned to baseline after 12 wk of WD, and was reduced below baseline levels after 24 wk of WD (Figure 2(h)). SIRP*α* and XCR1 gene expression was used to discriminate between CD11b^+^ cDC and CD103^+^ cDC, respectively [29]. The expression level of SIRP*α* increased 11-fold in mice after 12 wk of WD compared to mice sacrificed before the start of the WD (Figure 2(i)). A significant increase (2.6-fold) was also observed in the expression of XCR1 after 6 wk of WD (Figure 2(j)). The same is true for the amount of NKT cells, as detected by V*α*14-J*α*18 mRNA (2-fold increase, Figure 2(k)).

### 3.2. The Number of Circulating CD11b^+^ cDC and NKT Cells Is Highly Indicative for Plaque Inflammation in Mice during Atherosclerosis

**Figure 3:**
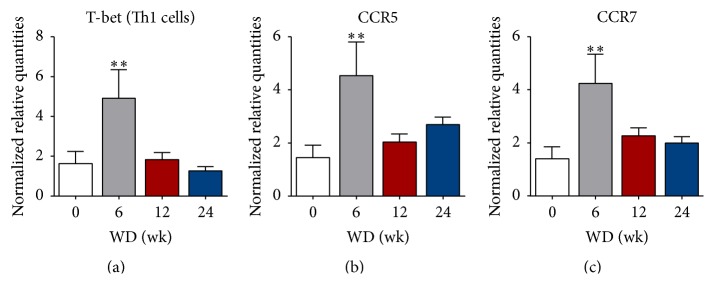
Features of inflammation in mouse aortic plaques. ((a), (b), (c)) Relative mRNA levels of T-bet (a), CCR5 (b), and CCR7 (c) in plaque-containing aortic tissue measured by real-time qPCR. 0 wk, *n* = 6, 6 wk, *n* = 5, 12 wk, *n* = 11, and 24 wk, *n* = 14-15; ^*∗*^ *P* < 0.05 and ^*∗∗*^ *P* < 0.01.

In mouse plaques, strong features of inflammation could be detected, including the expression of T-bet, the main director of Th1 lineage commitment [30]. The relative mRNA expression of T-bet was significantly increased (3-fold) after 6 wk of WD feeding compared to mice that had not yet received a WD (Figure 3(a)). Furthermore, the expression of different chemokine receptors, involved in homing of leukocytes to inflammatory sites or lymph nodes, was investigated. Plaque mRNA expression for C-C chemokine receptor type 5 (CCR5) and CCR7 was significantly higher (3-fold) after 6 wk of feeding on the WD and returned to baseline when lesions were progressing (Figures 3(b) and 3(c)). In aortic plaques, the expression of T-bet is positively correlated with CCR5 and CCR7 expression at 6 wk and 12 wk of WD (Table 3). Furthermore, there is a strong correlation between the degree of stenosis in the proximal aorta and the mRNA level of CCR7, V*α*14-J*α*18, Zbtb46, and SIRP*α* in mice sacrificed at 6 wk of WD. No correlations were found between the expression levels of CCR5/7 and the numbers of circulating T cells, CD4^+^ Th cells, B cells, or NK cells after 6 wk on the WD. In mice from the 12 wk of WD group, V*α*14-J*α*18 expression is correlated to T-bet, CCR5, CCR7, Zbtb46, SIRP*α*, and XCR1 (Table 3).

Because blood provides a conduit between all organs and tissues, correlations between local and circulating cells in atherosclerosis were also described using Spearman's rank-order correlation coefficient (Table 3). Plaque development and inflammation were most pronounced in mice that had a low number of circulating NKT cells at 6 wk of WD. At this time point, a strong inverse correlation was determined between NKT cell numbers in blood and the expression of T-bet, CCR5, CCR7, Zbtb46, and XCR1 in plaque-containing aortic tissue. In line with these findings, the degree of stenosis in the proximal aorta was inversely correlated to circulating NKT cell numbers. Similar observations were seen for the number of circulating CD11b^+^ cDC and the expression of the same genes in the aorta at 12 wk of WD.

### 3.3. CD11b^+^ cDC and NKT Cell Dynamics in ApoE^−/−^ Mice Are Distinct from Healthy Controls

**Figure 4:**
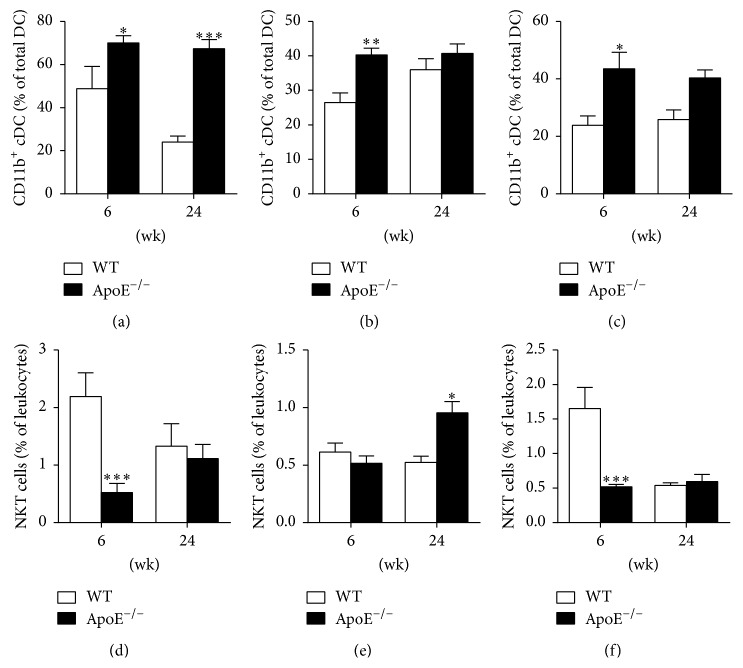
Comparison of CD11b^+^ cDC and NKT cell fluctuations between ApoE^−/−^ mice and age-matched healthy controls. (a–c) Percentages of circulating CD11b^+^ cDC in blood (a), spleen (b), and mediastinal LN (c) of ApoE^−/−^ mice fed a WD and healthy wild-type controls fed a chow diet sacrificed at 6 and 24 wk of diet. (d–f) Circulating NKT cell numbers in blood (d), spleen (e), and mediastinal LN (f) of ApoE^−/−^ mice fed a WD and healthy wild-type controls (on chow diet) sacrificed at 6 and 24 wk of diet. WT (white bars), *n* = 4–6; ApoE^−/−^ (black bars), *n* = 9–12; ^*∗*^ *P* < 0.05, ^*∗∗*^ *P* < 0.01, and ^*∗∗∗*^ *P* < 0.001.

To correct for changes related to age rather than atherosclerosis, we compared the number of CD11b^+^ cDC and NKT cells in ApoE^−/−^ mice fed an atherosclerotic diet and age-matched healthy wild-type mice fed a chow diet at two time points which represent early and advanced atherosclerotic lesions. Percentages of circulating CD11b^+^ cDC are higher in ApoE^−/−^ mice as compared to healthy wild-type controls in blood, spleen, and mediastinal LN during early lesion (6 wk of diet) formation (Figures 4(a)–4(c)). In the case of advanced plaques (24 wk of diet), the CD11b^+^ cDC percentage is also significantly higher in the blood of ApoE^−/−^ mice (Figure 4(a)). The opposite is seen for the NKT cells: at the initiation of the disease (6 wk of diet) NKT cell numbers in blood (Figure 4(d)) and mediastinal LN (Figure 4(f)) of ApoE^−/−^ mice are low as compared to healthy controls. There is no difference in the NKT cells percentage in the spleen between ApoE^−/−^ and control mice at the onset of atherosclerosis but they increase with enhanced atherosclerosis (Figure 4(e)). Hence, high numbers of CD11b^+^ cDC and low numbers of NKT cells at 6 wk of diet are attributable to the induction of atherogenesis in ApoE^−/−^ mice.

### 3.4. Analysis of Immune Cells in Human Atherosclerotic Plaques and Blood

**Figure 5:**
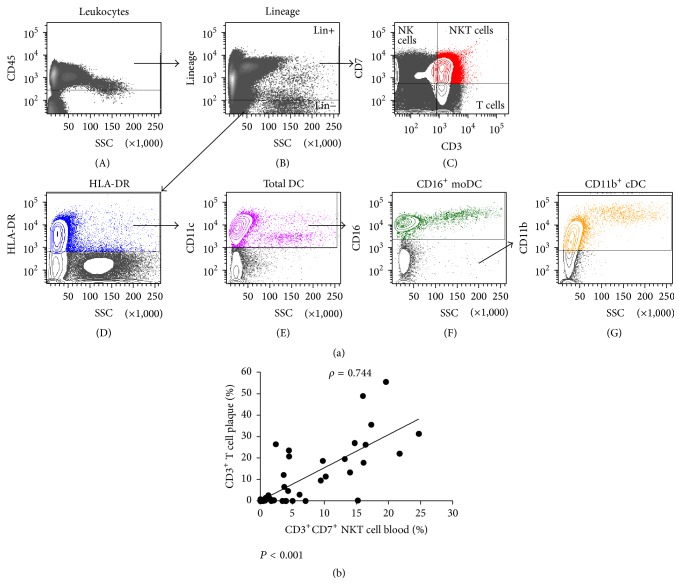
Leukocyte subsets and correlation analysis in human plaques and blood. (a) Gating strategy for the analysis of leukocyte(s) (subsets) in human plaque and blood samples. After staining the leukocyte population using a CD45 pan leukocyte antibody (A), lineage markers (= a CD3, CD14, CD19, CD20, and CD56 cocktail) were used to separate the DC (lineage−) from the other immune cells (lineage+) (B). Within the lineage+ cells we identified T cells, NK cells, and NKT cells, which were defined as CD3^+^, CD3^−^ CD7^+^, and CD3^+^ CD7^+^, respectively (C). cDC were then identified as positive for HLA-DR (D) and CD11c (E). CD16 was used for the staining of monocyte-derived DC (F). Subsequently, CD11b^+^ cDC (G) were gated from the CD16-negative population. Within the lineage+ population we identified monocytes in blood as HLA-DR^+^ CD11c^+^ CD11b^+^ CD14^+^ and macrophages in plaques as HLA-DR^+^ CD11c^+^ CD11b^+^ CD68^+^ (plots not shown). (b) Spearman's rank correlation plot showing the relationship between CD3^+^CD7^+^ NKT cell numbers in blood and the percentage of total CD3^+^ T cells in plaques obtained from the same endarterectomy patient (*n* = 57).

To analyze different leukocyte subsets in plaque and blood samples from advanced atherosclerosis patients, we used a gating strategy as depicted in Figure 5(a). As observed in mice and similar to our previous data [14] we observed predominance of CD11b^+^ cDC within the CD45^+^ population in the plaques, compared to the CD16^+^ monocyte-derived (mo)DC subset. In contrast to the plaque, the CD16^+^ moDC was the predominant subset in the blood compared to the CD11b^+^ cDC (Table 4). Clec9A was used as a marker for the human equivalent of CD103^+^ cDC in mice. However, due to their low numbers [14], we refrained from studying this cDC subset in subsequent analyses in this study. Within the CD45^+^ population, atherosclerotic plaques predominantly contained NK cells. Furthermore, relatively high mean percentages of NKT cells and T cells were also observed, both in blood and plaque, as compared to the DC (subsets) and monocytes/macrophages (Table 4).

To extend the evaluation of atherosclerotic plaque composition we compared the immune cell distribution in plaques from distinct anatomical locations (carotid versus femoral artery). To correct for size differences between plaques from femoral and carotid artery, the number of cells per gram tissue was calculated. Comparing between plaque locations, the number of cells per gram was significantly higher in carotid plaques for the total DC population (311 ± 70 versus 121 ± 24; *P* = 0.014), CD16^+^ moDC subset (20 ± 6 versus 5 ± 1; *P* = 0.029), CD11b^+^ cDC subset (224 ± 51 versus 85 ± 19; *P* = 0.014), NKT cells (1652 ± 623 versus 127 ± 38; *P* = 0.01), and T cells (1551 ± 556 versus 208 ± 65; *P* = 0.029). In contrast, the macrophage (403 ± 212 versus 10 ± 3; *P* = 0.260) and NK cell numbers per gram (1344 ± 221 versus 1092 ± 271; *P* = 0.477) did not significantly differ between the two locations.

### 3.5. In Advanced Human Atherosclerosis the Number of Circulating NKT Cells Is Predictive for Plaque Inflammation

We investigated whether correlations could be found between the immunological parameters in blood and advanced plaques collected from the same endarterectomy patient. We could find a strong predictive role for NKT cells which strengthens our observations in mice. Remarkably, the percentage of NKT cells in blood correlates strongly with the percentage of total T cells in the plaque (*ρ* = 0.744; *P* < 0.001) (Figure 5(b)). A linear regression analysis was performed to explore which of the human plaque variables can be predicted by blood variables. In all models, the logarithm of the plaque variables was entered as outcome variable. Here, we could see that the predictive value of the percentage of NKT cells in blood to predict the T cell (and NKT cell) load in the plaque is very strong (*P* < 0.001). This was also the case for the prediction of the NK cell and macrophage load (*P* < 0.05). Subsequently, for each of the plaque variables, a multiple linear regression model was fitted with all the risk and blood parameters as independent variables. For these models, the coefficient of determination (*R* ^2^) was calculated (Table 5). This shows that T cells (*R* ^2^ = 0.802104), NK cells (*R* ^2^ = 0.744789), and NKT cells (*R* ^2^ = 0.727062) in plaques are predictable by the combination of risk factors and blood variables. Next, stepwise backward model building was performed, starting with a model including all the plaque variables with *R* ^2^ > 0.6, to obtain multiple regression models with only the most significant predictors for each plaque variable. Strikingly, for both the percentages of T cells and NKT cells in plaques, the NKT cell numbers in blood are the most significant predictors. In addition, partial *R* ^2^ values were calculated to describe how strongly the cells in blood contribute to the prediction of cells in the plaque, based on all blood parameters and risk factors from a patient. The NKT cell numbers in blood strongly improve the prediction of the amount of both NKT cells (partial *R* ^2^ = 0.36; *P* = 8.9 × 10^−9^) and T cells (partial *R* ^2^ = 0.20; *P* = 1.1 × 10^−7^) in plaques, even if all other risk factors are accounted for. For the prediction of NK cells in plaques, the contribution of NKT cells in blood was not significant (partial *R* ^2^ = 0.006; *P* = 0.34).

C-reactive protein (hs-CRP) has been endorsed by multiple guidelines as a biomarker of atherosclerotic cardiovascular disease risk [31, 32]. However, in this study, hs-CRP levels in blood do not correlate with the percentages of NKT cells in blood or plaques from the same patient (data not shown), although this may be due to the fact that there were only few data points available for hs-CRP (*n* = 35).

## 4. Discussion

To date, only a few studies reported the analysis and association of circulating inflammatory cells and advanced atherosclerosis. Most of the existing data come from subclinical atherosclerosis and asymptomatic patients [33]. The aim of the present study was to analyze the frequency of immune cells in blood, plaque, and associated lymphoid tissues (i.e., mouse spleen and aorta-draining LN) and to investigate whether fluctuations in leukocytes are associated with or can be predictive for plaque growth and inflammation.

The most pronounced changes during atherosclerosis in mice occur early in plaque development in cells of the innate immune system. Early atherogenesis is marked by an elevation in plasma cholesterol levels followed by (oxidative) modification of low density lipoproteins, a well-known trigger of inflammation. Antigen-presenting cells are needed at this time to encounter these “foreign” antigens; hence more DC are present in the circulation and draining lymph nodes. As atherosclerosis progresses the number of CD11b^+^ cDC declines significantly at 12 wk of WD in all locations investigated, suggestive of massive recruitment to the growing lesions in the aortic wall. Indeed, we observed an increase in the relative expression level of SIRP*α* in the aorta at the same time, together with a substantial increase in plaque size in the brachiocephalic artery and proximal ascending aorta. Recruitment of immune cells to sites of inflammation, infection, or injury is stimulated by chemokines and their receptors [34–37]. CCR5 directs recruitment of immune cells to inflammatory sites like atherosclerotic lesions, while CCR7 can mediate monocyte/macrophage egress from lesions and controls the subsequent migration of immune cells from the plaque to secondary lymphoid organs [37]. We observed a strong inverse correlation between circulating CD11b^+^ cDC numbers and CCR5/7 expression in mouse aortic plaques at 12 wk of WD. Accordingly, mice that have a low number of CD11b^+^ cDC in their circulation, as is the case at 12 wk of WD, have high expression levels of CCR5/7 in their plaques. This indicates a high degree of leukocyte trafficking to and from the plaque. In addition, we have also seen an inverse correlation between circulating CD11b^+^ cDC and the expression levels of T-bet, V*α*14-J*α*18, and Zbtb46, which points to an increased inflammatory status in the plaque.

Additionally, this study revealed that plaque development and inflammation were most pronounced in mice that have a low number of circulating NKT cells at 6 wk of WD. At this time point, expression of inflammation markers, including T-bet, chemokines (CCR5/7), and cDC (Zbtb46), as well as the degree of stenosis in the proximal ascending aorta, correlated with NKT cell numbers in blood, pointing to a very significant role of NKT cells in the initiation of atherosclerosis. In line with these findings, previous research demonstrated that the contribution of NKT cells on atherosclerosis is transient and limited to early fatty streak lesions [38, 39]. Similar to the observations made by Aslanian et al., we detected V*α*14-J*α*18 mRNA in early lesions (6 wk of WD) but found no accumulation of V*α*14-J*α*18 after the 6-week time point (12 and 24 wk of WD) [38]. Consistent with results from a study by Major et al., we found that NKT cell numbers are low in blood and LN of ApoE^−/−^ mice compared with age-matched wild-type mice at an early stage of atherosclerosis development (6 wk of diet) [40]. As the lesion progresses to advanced atherosclerosis, the total DC number increased in the spleen after 12 wk and even more after 24 wk on the WD, due to systemic immune activation [41]. Additionally, this could also be the result of extramedullary hematopoiesis. When the bone marrow can no longer handle the production and differentiation of hematopoietic cells, it will outsource the production of circulating leukocytes, including DC. Of all organs, the spleen is an ideal outsource destination in ApoE^−/−^ mice [42, 43].

Taken together, these data suggest that inflammatory processes, with an emphasis on CD11b^+^ cDC and NKT cells, are crucial in the early development of atherosclerosis before any morphological changes (plaque development) are visible. Based on these data, we propose 12 wk as a preferred time point for intervention, especially when assessing the effects of immunomodulatory therapies for preventing the development and progression of atherosclerosis. This is in agreement with Jeon et al., who reported a peak at 12 wk of diet in inflammatory mediators ICAM-1, CCR2, IL-6, IL-12p40, and IL-17 [41].

Parallel to mice, we also observed predominance of the CD11b^+^ cDC subset in human plaques when compared with CD16^+^ moDC, while the latter is the main subset in blood. In a recent study, CD11b^+^ cDC were described to promote atherosclerosis development by limiting the expansion of Tregs [44]. In consonance with the drop in circulating CD11b^+^ cDC in mice, we and others have shown previously that circulating CD11b^+^/BDCA-1^+^ cDC numbers are reduced in patients with coronary artery disease [45, 46]. In this study, we only enrolled patients with symptomatic advanced atherosclerosis and were therefore not able to draw a comparison with asymptomatic patients. However, to our knowledge, we are the first to report a direct correlation between NKT cell numbers in blood and the load of T cells (and NKT cells) in the atherosclerotic plaques. Both CD1d expressing cells and NKT cells were previously shown to be present in advanced human atherosclerotic plaques [47]. Here, we demonstrated that the percentage of NKT cells in blood strongly improves the prediction of both T cells and NKT cells in the plaque, independent of all the other risk factors. In line with these findings, Levula et al. applied gene set enrichment analysis and real-time qPCR to human advanced atherosclerotic plaques from carotid and femoral arteries as well as aortas. 26 genes, out of a total of 29 genes, of the NKT pathway were significantly upregulated in atherosclerotic plaques versus nonatherosclerotic controls [48]. Furthermore, in humans, it was reported that circulating NKT cell numbers are reduced in patients who experienced previous cardiovascular events compared with either asymptomatic atherosclerosis patients or young healthy individuals [47]. Unfortunately, data on leukocyte cell numbers in the arterial wall during early atherogenesis are virtually nonexistent as patients mostly present themselves in the clinic when serious blockages are already present. Nevertheless, in the search for better or additional biomarkers that can alert physicians for the presence of inflammatory plaques, circulating NKT cells should be further explored, as also proposed for type 2 diabetes and cancer [49, 50]. We need to, however, remain cautious. Even in healthy individuals NKT cell numbers can fluctuate substantially and NKT cell subsets may play different functional roles in atherosclerosis [51]. Future studies, including a higher number of patients and different stages of atherosclerosis, will need to clarify the true potential of NKT cells as biomarkers (or even cellular therapy) for inflammatory, and thus unstable, atherosclerotic plaques.

Finally, we could not find a correlation between circulating hs-CRP levels and the percentages of NKT cells in blood or plaques, although this is most likely due to the small sample size and the associated large variation in hs-CRP levels. CRP is increased in individuals with an overlap to other risk factor pathways such as obesity, low social class, and smoking. Studies on the added value of CRP in risk prediction of cardiovascular disease show that hs-CRP levels can confirm the presence of plaques but do not provide insight on the degree of stenosis or the inflammation in the plaque [33].

To date, the value of circulating leukocyte profiles as biomarker of atherosclerosis is underappreciated [33]. Despite the fact that cDC and NKT cells are quantitatively minor components of the immune system, they do appear to play a major role in modulating the course of the disease. We believe that a profound analysis of circulating leukocytes, in particular CD11b^+^ cDC and NKT cells, may thus provide a helpful tool to assess the inflammatory and immune status of an atherosclerosis patient.

## 5. Conclusion

We provide an extensive quantitative description of systemic and peripheral immune cell dynamics over the entire life span of atherosclerotic lesion development in ApoE^−/−^ mice. Based on the crucial shift in leukocyte trafficking at 12 wk of WD, we propose this to be a preferred time point for therapeutic intervention, aimed at targeting the dysregulated immune response in atherosclerosis. Furthermore, our results show that circulating NKT cells may carry biomarker potential reflecting atherosclerotic lesion progression and/or inflammation, both in mice and humans. Because of its predictive value, the DC-NKT cell axis in atherosclerosis could provide potential as a tool for better patient risk stratification and/or a target for plaque stabilization, especially when determining the optimal timing for therapy.

## Supplementary Material

Table S1: Monoclonal antibodies used for the flow cytometric analysis in this study.
Table S2: Primer sequences for PCR.

## Figures and Tables

**Table 1:** Patient characteristics.

Variable	Carotid artery plaque (*n* = 35)	Femoral artery plaque (*n* = 37)	Significance^†^
Age (years)	72 ± 2	71 ± 2	NS
Male gender (%)	60	68	NS
Degree of artery stenosis (%)	82 ± 2	87 ± 1	NS
Risk factors (%)			
Family history	20	46	*P* < 0.05^*∗*^
Hypertension	71	78	NS
Hypercholesterolemia	80	75	NS
Diabetes mellitus	42	27	NS
Smoking	46	70	*P* < 0.01^*∗∗*^
Obesity	29	22	NS
Prior vascular intervention	46	68	NS
Medication (%)			
Acetylsalicylic acid	98	92	NS
NSAIDs	3	3	NS
Beta-blockers	46	65	NS
Calcium channel blockers	34	22	NS
ACE inhibitors	43	35	NS

^†^Significant differences between plaque locations; NS: no significance.

**Table 2:** Cholesterol and plaque parameters of ApoE^−/−^ mice during atherogenesis.

	0 wk	6 wk	12 wk	24 wk
Cholesterol (mg/dL)	232 ± 11	625 ± 97^*∗∗∗*^	658 ± 40^*∗∗∗*^	698 ± 45^*∗∗∗*^
Stenosis *A*_prox_ (%)	0 ± 0	1.2 ± 0.4	13.8 ± 2.5^*∗∗∗*^	23.2 ± 2.3^*∗∗∗*^
Stenosis *A*_br_ (%)	0.8 ± 0.8	1.8 ± 1.1	51.3 ± 7.7^*∗∗∗*^	61.5 ± 2.8^*∗∗∗*^
Necrotic core *A*_prox_ (%)	0 ± 0	0 ± 0	0 ± 0	3.5 ± 1.1^*∗∗*^
Necrotic core *A*_br_ (%)	0 ± 0	0 ± 0	1.4 ± 0.7	5.5 ± 1.3^*∗∗∗*^

Data from proximal ascending aorta (*A*_prox_) and brachiocephalic artery (*A*_br_), mean ± SEM, 0 wk, *n* = 9–11, 6 wk, *n* = 11-12, 12 wk, *n* = 10-11, and 24 wk, *n* = 11-12; ^*∗∗*^ *P* < 0.01 and ^*∗∗∗*^ *P* < 0.001.

**Table 3:** Spearman's rank correlation coefficients (*ρ*) of associations between gene expression results within plaques or between plaques and circulating NKT cells or CD11b^+^ cDC.

6 wk of WD								
	T-bet	CCR5	CCR7	V*α*14-J*α*18	Zbtb46	SIRP*α*	XCR1	Stenosis
T-bet^(p)^	—	0.900	0.700	0.200	−0.200	−0.400	0.900	0.354
Stenosis^(p)^	0.354	0.354	0.707	0.707	0.755	0.755	0.354	—
V*α*14-J*α*18^(p)^	0.200	0.500	0.300	—	0.400	0.200	0.500	0.707
NKT cells^(b)^	−1.000	−0.800	−1.000	−0.400	−1.000	−0.500	−0.800	−0.657
CD11b^+^ DC^(b)^	−0.400	0.000	−0.100	0.500	0.000	0.000	0.000	−0.251

12 wk of WD								
	T-bet	CCR5	CCR7	V*α*14-J*α*18	Zbtb46	SIRP*α*	XCR1	Stenosis

T-bet^(p)^	—	0.855	0.855	0.891	0.758	−0.818	0.952	−0.261
Stenosis^(p)^	−0.261	−0.515	−0.393	−0.370	−0.381	0.345	−0.200	—
V*α*14-J*α*18^(p)^	0.891	0.721	0.879	—	0.636	−0.830	0.939	−0.370
NKT cells^(b)^	−0.456	−0.535	−0.426	−0.322	−0.116	0.274	−0.377	−0.189
CD11b^+^ DC^(b)^	−0.717	−0.733	−0.717	−0.583	−0.650	0.633	−0.733	0.400

p, plaque; b, blood.

The magnitude of the correlation coefficient determines the strength of the correlation: |*ρ*| > 0.7 strong correlation; 0.5 < |*ρ*| < 0.7 moderate correlation; |*ρ*| < 0.5 weak correlation.

**Table 4:** Flow cytometric analysis of immune cells in human atherosclerotic plaques and blood.

	Blood (% within CD45^+^ population)	Plaque (% within CD45^+^ population)
Total DC		
CD11c^+^ DC	6.5 ± 0.9	2.0 ± 0.2
DC subsets		
CD11b^+^ cDC	2.7 ± 0.4	1.4 ± 0.2
CD16^+^ moDC	3.7 ± 0.5	0.3 ± 0.1
Other leukocytes		
CD14^+^ Mo	0.4 ± 0.1	NA
CD68^+^ M*φ*	NA	1.1 ± 0.8
CD3^+^ T cell	10.3 ± 1.8	5.9 ± 1.0
CD7^+^ NK cell	9.5 ± 1.7	14.1 ± 1.3
CD3^+^CD7^+^ NKT cell	8.9 ± 1.8	6.7 ± 1.5

Mo, monocyte; M*φ*, macrophage; NA, not applicable.

**Table 5:** Coefficient of determination (*R* ^2^) values for each of the plaque variables calculated from a multiple linear regression model with all the risk factors and blood parameters as independent variables (*n* = 57 plaque and blood samples). This is the amount of variance in the outcome (plaque variable) that can be explained by all the risk factors and blood variables together.

Plaque variable (%)	*R* ^2^
Total DC	0,484670
CD11b^+^ cDC	0,434602
CD16^+^ moDC	0,534353
Macrophages	0,500945
T cell	0,802104
NK cells	0,744789
NKT cells	0,727062
