# Supplementary material for: Linking CD11b+ Dendritic Cells and Natural Killer T Cells to Plaque Inflammation in Atherosclerosis
Source: Mediators Inflamm. 2016 Mar 9;2016:6467375. doi: 10.1155/2016/6467375 (PMC4804096; doi:10.1155/2016/6467375)
Supplement: Supplementary file 1 — Table S1: Monoclonal antibodies used for the flow cytometric analysis in this study. Table S2: Primer sequences for PCR. [file 6467375.f1.docx]

**Supplementary Material**

**Table S1. Monoclonal antibodies used for the flow cytometric analysis in this study.**

| **Marker** | **Conjugate** | **Clone** | **Specificity** | **Source** |
| --- | --- | --- | --- | --- |
| **Anti-mouse antibodies** | | | | |
| CD11c | APC | N418 | cDC | BioLegend |
| I-A^b^ | FITC | KH74 | cDC, B cells | BioLegend |
| CD11b | PerCP | M1/70 | cDC2, macrophages | BioLegend |
| CD103 | PerCP-Cy5.5 | 2E7 | cDC1 | BioLegend |
| CD3ε | APC | 145-2C11 | T cells | BioLegend |
| CD4 | PerCP | GK1.5 | Th cells | BioLegend |
| CD19 | PE | 6D5 | B cells | BioLegend |
| NK1.1 | FITC | PK136 | NK(T) cells | BioLegend |
| Ly6C | APC | HK1.4 | Monocytes | BioLegend |
| CD68 | APC | FA-11 | Macrophages | BioLegend |
| **Anti-human antibodies** | | | | |
| CD45 | APC-H7 | 2D1 | Leukocytes | BD |
| Lineage 2 | FITC | SK7 (CD3), SJ25C1 (CD19), L27 (CD20), MΦP9 (CD14),  NCAM16.2 (CD56) | T cells, monocytes, macrophages, neutrophils, eosinophils, B cells, NK cells | BD |
| HLA-DR | V500 | G46-6 | Total DC | BD |
| CD11c | PE-CF594 | B-ly6 | cDC | BD |
| CD11b | BV421 | ICRF44 | BDCA-1^+^ cDC | BD |
| CD16 | PerCP-Cy5.5 | 3G8 | moDC | BD |
| CD3 | PE-Cy7 | SP34-2 | T cells, NKT cells | BD |
| CD7 | PE | M-T701 | NK(T) cells | BD |
| CD14 | APC | M5E2 | Macrophages, monocytes | BD |
| CD68 | APC | Y1/82A | Macrophages, monocytes | BioLegend |

Abbreviations: BDCA-1, blood dendritic cell antigen-1; IDC, (conventional) dendritic cells; HLA-DR, human leukocyte antigen; I-A^b^, major histocompatibility complex class II alloantigen; moDC, monocyte-derived dendritic cells; NK(T) cells, natural killer (T) cells; Th, T-helper cells.

**Table S2. Primer sequences for PCR.**

| **Gene** | **Sequence sense** | **Sequence antisense** |
| --- | --- | --- |
| **Genes of interest** | | |
| **CCR5** | CAAGACAATCCTGATCGTGCAA | TCCTACTCCCAAGCTGCATAGAA |
| **CCR7** | AGAAGAACAGCGGCGAGGA | AGCATAGGCACTAGGAACCCAAA |
| **SIRPα** | ATACGCAGACCTGAATGTGCCCAA | TGGCCACTCCATGTAGGACAAGAA |
| **T-bet** | GCCAGGGAACCGCTTATATG | GACGATCATCTGGGTCACATTGT |
| **Vα14-Jα18** | TGGGAGATACTCAGCAACTCTGG | CAGGTATGACAATCAGCTGAGTCC |
| **XCR1** | CATGGGTTCTTGGCCTCAGT | ACAGTGCTGGATGTCTTCCG |
| **Zbtb46** | TCACATACTGGAGAGCGGC | CCTCATCCTCATCCTCAACC |
| **Housekeeping genes** | | |
| **GAPDH** | CCAGTATGACTCCACTCACG | GACTCCACGACATACTCAGC |
| **PPIA** | GAAGCCATGGAGCGTTTTGG | CAGATGGGGTAGGGACGCTC |

Abbreviations: CCR5/7, C-C chemokine receptor type 5/7; GAPDH, glyceraldehyde-3-phosphate dehydrogenase; PPIA, peptidylprolyl isomerase A; SIRPα, signal regulatory protein α; T-bet, T-box transcription factor expressed in T cells; Vα14-Jα18, invariant TCR-α chain rearrangement specific for NKT cells; XCR1, chemokine XC receptor 1.
